# Changes in tubal permanent contraception rates in South Carolina following the *Dobbs* decision: a retrospective cohort study

**DOI:** 10.3389/frph.2026.1946711

**Published:** 2026-09-23

**Authors:** Isabella Mateu, Erica Lowing, Jessica Britt, Erica F. Robinson

**Affiliations:** Department of Obstetrics and Gynecology, Prisma Health Upstate, University of South Carolina School of Medicine, Greenville, SC, United States

**Keywords:** abortion access, *Dobbs v Jackson*, permanent contraception, roe v wade, South Carolina, sterilization

## Abstract

**Background:**

The Supreme Court finalized its decision in *Dobbs v Jackson Women's Health Organization* on June 24, 2022, overturning nearly 50 years of constitutional protection for abortion in the United States under *Roe v Wade* (1973) and *Planned Parenthood v Casey* (1992). This subsequently allowed states to implement or enforce more restrictive abortion policies and bans. Multiple studies have demonstrated increased demand for permanent contraception at the national level and across institutions in states with varying degrees of abortion policies. Whether a similar increase occurred in South Carolina, one of the most restrictive states with regards to access to abortion care, has not yet been described and is the focus of this study.

**Objective:**

We aim to assess the potential impact of the *Dobbs* decision on tubal permanent contraception by comparing the proportion of patients undergoing these procedures at a large multi-hospital system in South Carolina before and after the Supreme Court ruling.

**Study design:**

This observational, retrospective cohort study included 141,375 reproductive-age patients who were active at a large multi-hospital health system between June 24, 2021 and June 25, 2023. The primary outcome was the proportion of active patients undergoing sterilization in the year following the Supreme Court's ruling (June 2022-June 2023, “Post-*Dobbs”*) compared to the year preceding the ruling (June 2021-June 2022, “Pre-*Dobbs”*). Secondary outcomes included demographic measures to assess how factors differed between those who did and did not receive permanent contraception. Statistical analysis included chi square and t-tests, as appropriate, and multivariable logistic regression was used to estimate adjusted odds of sterilization.

**Results:**

The proportion of patients undergoing permanent contraception was 2.34% in the “Post-*Dobbs*” period compared to 1.83% in the “Pre-*Dobbs*” period, representing an absolute increase of 0.51%. Patients in the “Post-*Dobbs”* period had higher odds of undergoing sterilization compared to the “Pre-*Dobbs”* period [OR 1.28 (95% CI 1.19-1.38); adjusted OR 1.24 (95% CI 1.14-1.35); adjusted for race, ethnicity, healthcare payor, marital status, parity, gravidity, and maternal age]. The proportion increased from 1.52% in the six months prior to the *Dobbs* decision to 1.83% in the six months following the decision (*p* = 0.0006) and further increased to 2.12% in the subsequent six months (*p* = 0.002). Additionally, demographic differences were observed between patients who did and did not receive permanent contraception.

**Conclusion:**

The proportion of patients undergoing permanent contraception increased following the *Dobbs* decision and continued to rise for at least one year. These findings suggest an association between increased abortion restrictions and increased utilization of permanent contraception.

**Implications:**

These findings suggest an association between restrictive abortion policies and increased utilization of permanent contraception in South Carolina, with continued rise over the subsequent year. Demographic shifts among patients undergoing sterilization warrant continued monitoring, particularly as funding and state abortion policies continue to evolve.

## 1 Introduction

After an initial draft was leaked on May 2, 2022, the US Supreme Court finalized its decision in *Dobbs v Jackson Women's Health Organization* on June 24, 2022. This ruling reversed the nearly 50-year constitutional right to abortion in the United States previously protected under *Roe v Wade* (1973) and *Planned Parenthood v Casey* (1992), subsequently allowing states to enact varying degrees of abortion restrictions (1). In South Carolina specifically, several rulings passed through courts during the post-*Dobbs* period and ultimately in May of 2023, the governor signed into law S.474, the “Fetal Heartbeat and Protection from Abortion Act.” This law essentially banned the termination of any intrauterine pregnancy with a heartbeat except under a few designated exceptions, including rape and incest up to 12 weeks gestation, lethal fetal anomaly, and to prevent maternal death or severe morbidity (2). The South Carolina Supreme Court upheld this legislation in August 2023 (Figure 1).

**Figure 1:**
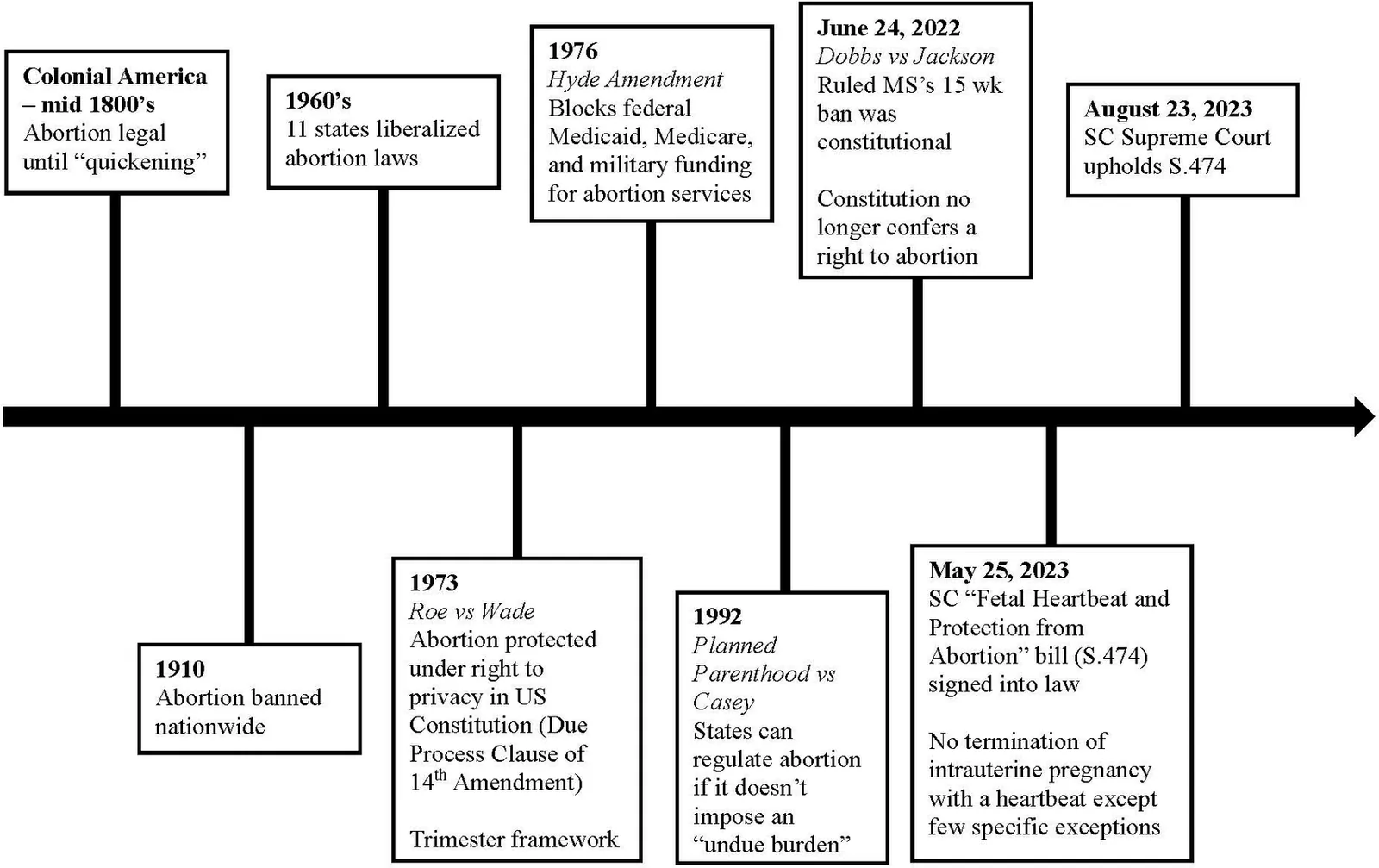
Legislative changes regarding abortion restriction over time at the national and state level.

Following the *Dobbs* decision, multiple studies have demonstrated increased demand for permanent contraception at the national level and across institutions in states with varying abortion policies (3–10). The focus of this study is to determine if a similar increase occurred in South Carolina. While it does not have a total abortion ban, this state is one of eleven whose government has enacted laws to prohibit access to abortion care, where abortion is prohibited after detection of fetal cardiac activity with few exceptions (11).

In this study, we aim to assess the potential impact of the *Dobbs* decision on tubal permanent contraception in South Carolina by comparing changes in the frequency of these procedures prior to and following the *Dobbs* ruling.

## 2 Materials and methods

This was an observational, retrospective cohort study conducted at the largest multi-hospital health system in South Carolina. Hospital-based clinics and associated primary OBGYN groups in the Upstate and Midlands of South Carolina were included in the study. The study was reviewed and approved by the Prisma Health institutional review board (IRB approval #2166566). The population of interest included reproductive age patients who were active at an OBGYN practice in our system between June 24, 2021 and June 25, 2023. Inclusion criteria were patients assigned female sex at birth and age 21 to 50 at time of inclusion into the study to include all patients who were potentially eligible for permanent contraception and to exclude patients receiving routine care outside of general reproductive age. Patients undergoing a sterilization procedure were identified by CPT codes and confirmed with verification of an ICD-10 code for sterilization or contraception directly associated with the procedure [A].

The primary outcome was the proportion of active patients undergoing sterilization procedures within a large multi-health hospital system serving over 25% of the state population. This proportion was compared between the year following the Supreme Court's ruling (June 25, 2022 to June 25, 2023, titled “Post-*Dobbs*”) to the year preceding the overturning (June 24, 2021 to June 24, 2022, titled “Pre-*Dobbs*”). Patients were considered active if they had at least one in-person office visit, initial prenatal visit, routine prenatal visit, or clinical support encounter during the study period. Because the health system experienced substantial growth during this time, including the acquisition of additional clinical practices, the number of patients receiving care increased over time. As a result, changes in procedural counts alone could reflect shifts in patient volume rather than true differences in utilization. To address this, we evaluated sterilization both as absolute procedural volume and as a proportion of active patients, thereby accounting for changes in the size of the underlying population.

The proportion (i.e., cumulative risk) of sterilization was defined as:

$$Proportion\;of\;sterilization=\frac{number\;of\;sterilization\;procedures}{number\;of\;active\;patients}$$

Patients could contribute to both the “Pre-*Dobbs”* and “Post-*Dobbs”* populations if they remained active across both periods. However, patients who underwent sterilization during the “Pre-*Dobbs*” timeframe were excluded from the “Post-*Dobbs*” denominator, as they were no longer eligible for the procedure (*n* = 331). For patients with multiple encounters and varying sociodemographic characteristics (e.g., age, marital status, gravidity, parity, healthcare payor), a single encounter was randomly selected within each period for analysis. Sterilization proportions were also calculated on a six-month basis. For the primary “pre-*Dobbs*” vs. “post-*Dobbs*” analysis, each patient contributed a maximum of one observation to the “pre-*Dobbs”* cohort and one observation to the “post-*Dobbs”* cohort. Patients who remained active during both periods could therefore contribute to both cohorts. Sterilization proportions from this analysis reflect unique patients undergoing sterilization before and after the *Dobbs* decision. For analyses examining six-month trends, patients were eligible to contribute one observation within each six-month analytic interval. As a result, patients who remained active throughout the study period could contribute up to four observations. This approach allowed evaluation of temporal trends while accounting for changes in the size and demographic composition of the active patient population over time.

Secondary outcomes included demographic characteristics, including age, race, parity, marital status, and healthcare payor to assess how demographic factors differed between those who did and did not receive permanent contraception. All demographic factors were drawn from categorical data within the electronic health record. Notably, marital status was recorded according to legally documented electronic health record classifications, rather than cohabitation or partnership status. Additionally, race and ethnicity were based on patient self-report within the electronic health record. Both factors were recorded as separate variables, so patients identifying as Hispanic ethnicity could concurrently select any racial category. Sociodemographic factors were compared between the “Pre-*Dobbs*” and “Post-*Dobbs*” groups using chi square and t-tests as appropriate. Descriptive statistics included mean, standard deviation, frequency, and percentage.

The primary outcome was analyzed using multivariable logistic regression to compare the odds of sterilization in the “Post-*Dobbs”* versus “Pre-*Dobbs”* periods, adjusting for race, ethnicity, healthcare payor, marital status, parity, gravidity, and age. Chi-squared tests were used to compare differences in proportions across approximately six-month intervals throughout the study period. For the secondary analyses, demographic characteristics were compared between patients who received a sterilization procedure and those that did not using chi-square and t-tests as appropriate. All analyses were conducted using SAS (version 8.3; SAS Institute, Cary, NC). Statistical significance was determined as *p* < 0.05.

## 3 Results

The study population included 141,375 active patients, with 67,375 seen between June 24, 2021 and June 24, 2022 (the “Pre-*Dobbs*” group) and 74,000 seen between June 25, 2022 and June 25, 2023 (the “Post-*Dobbs*” group) (Table 1). Of the 106,569 unique patients included in the study, 34,788 contributed observations during both the “Pre-*Dobbs*” and “Post-*Dobbs*” periods. Race, ethnicity, marital status, healthcare payor, parity, and gravidity differed between groups (*p* < 0.01); however, age did not. Compared to the “Pre-*Dobbs”* group, the “Post-*Dobbs*” group had a higher percentage of Black (28.5% vs. 25.7%), Hispanic/Latinx (7.5% vs. 7.1%), and single patients (42.2% vs. 39.2%), as well as more patients with a government healthcare payor (25.6% vs. 22.5%). Additionally, mean parity [1.5 (1.3) vs. 1.4 (1.3)] and gravidity [2.0 (1.8) vs. 1.9 (1.8)] were slightly higher in the “Post-*Dobbs*” group (*p* < 0.0001) (Table 1).

**Table 1:** Demographic characteristics nof reproductive-age patients active in obstetrics and gynecology care at a multi-hospital health system in South Carolina, before and after the *dobbs* decision (June 2021-June 2023).

Characteristic	All	Pre-*Dobbs* (before June 25th, 2022)	Post-*Dobbs* (on or after June 25th, 2022)	*P*- Value
	141,375	67,375	74,000	
Age, years, mean (SD)	34.4 (8.2)	34.4 (8.2)	34.4 (8.2)	0.88
Race/Ethnicity				<0.0001
Black	27.1 (37,738)	25.7 (17,073)	28.5 (20,665)	
Hispanic	6.0 (8,352)	5.7 (3,789)	6.3 (4,563)	
White	62.4 (86,712)	64.2 (42,760)	60.6 (43,952)	
Other	4.5 (6,249)	4.4 (2,952)	4.6 (3,297)	
Ethnicity, Hispanic	7.3 (10,194)	7.1 (4,715)	7.5 (5,479)	0.001
Marital Status				<0.0001
Single	40.7 (55,985)	39.2 (25,951)	42.2 (30,034)	
Married	52.5 (72,210)	54.0 (35,788)	51.1 (36,422)	
Life Partner	0.7 (987)	0.7 (437)	0.8 (550)	
Divorced	5.7 (7,797)	5.8 (3,856)	5.5 (3,941)	
Other	0.4 (511)	0.4 (243)	0.4 (268)	
Healthcare Payor				<0.0001
Commercial	70.4 (99,567)	71.9 (48,470)	69.1 (51,097)	
Government	24.1 (34,115)	22.5 (15,173)	25.6 (18,942)	
Self-Pay/Uninsured	5.1 (7,163)	5.2 (3,509)	4.9 (3,654)	
Other	0.4 (530)	0.3 (223)	0.4 (307)	
Parity, mean (SD)	1.4 (1.3)	1.4 (1.3)	1.5 (1.3)	<0.0001
Gravidity, mean (SD)	2.0 (1.8)	1.9 (1.8)	2.0 (1.8)	<0.0001

During the “Pre-*Dobbs*” period, there were 1,213 sterilizations and 66,162 active patients. During the “Post-*Dobbs”* period there were 1,693 sterilization procedures and 72,307 active patients. The overall proportion of active patients undergoing sterilization was 2.34% in the “Post-*Dobbs*” period compared to 1.83% in the “Pre-*Dobbs*” period, corresponding to a modest absolute increase of 0.51%. This translates to 24% higher adjusted odds of undergoing sterilization in the “Post-*Dobbs”* period compared to the “Pre-*Dobbs”* period [OR 1.28 (95% CI 1.19-1.38); adjusted OR 1.24 (95% CI 1.14-1.35)] when adjusted for race, ethnicity, healthcare payor, marital status, parity, gravidity, and maternal age (Table 2). When examined in six-month intervals, proportion of sterilization procedure counts increased over time following *Dobbs*. During the “Pre-*Dobbs”* period, the proportion of active patients who underwent sterilization was 1.56% between June 24, 2021 and December 31, 2021 and 1.52% January 1, 2022 and June 24, 2022, reflecting a slight non-significant decrease (*p* = 0.70). In contrast, during the “Post-*Dobbs”* period, the proportion of sterilization procedures increased to 1.83% between June 25, 2022 and December 31, 2022 (*p* = 0.0006) and further increased to 2.12% between January 1, 2023 and June 25, 2023 (*p* = 0.002) (Table 3, Figure 2).

**Table 2:** Proportion of reproductive-age patients undergoing sterilization procedures before and after the *dobbs* decision at a multi-hospital health system in South Carolina (June 2021-June 2023).

Time frame	Pre-*Dobbs*	Post-*Dobbs*	OR (95% CI)	aOR (95% CI)
Proportion of Sterilization^a^	1.83 (1,213/66,162)	2.34 (1,693/72,307)	1.28 (1.19, 1.38)	1.24 (1.14, 1.35)

a Adjusted for race, ethnicity, healthcare payor, marital status, parity, gravidity, and maternal age.

**Table 3:** Six-month trends in the proportion of sterilization procedures among reproductive-age patients at a multi-hospital health system in South Carolina (June 2021-June 2023).

Time frame	Proportion	*p*-value (change from previous)
6/24/21–12/31/21	1.56 (585/37,593)	
1/1/22–6/24/22	1.52 (628/41,318)	0.70
6/25/22–12/31/22	1.83 (868/47,404)	0.0006
1/1/23–6/25/23	2.12 (825/38,988)	0.002

**Figure 2:**
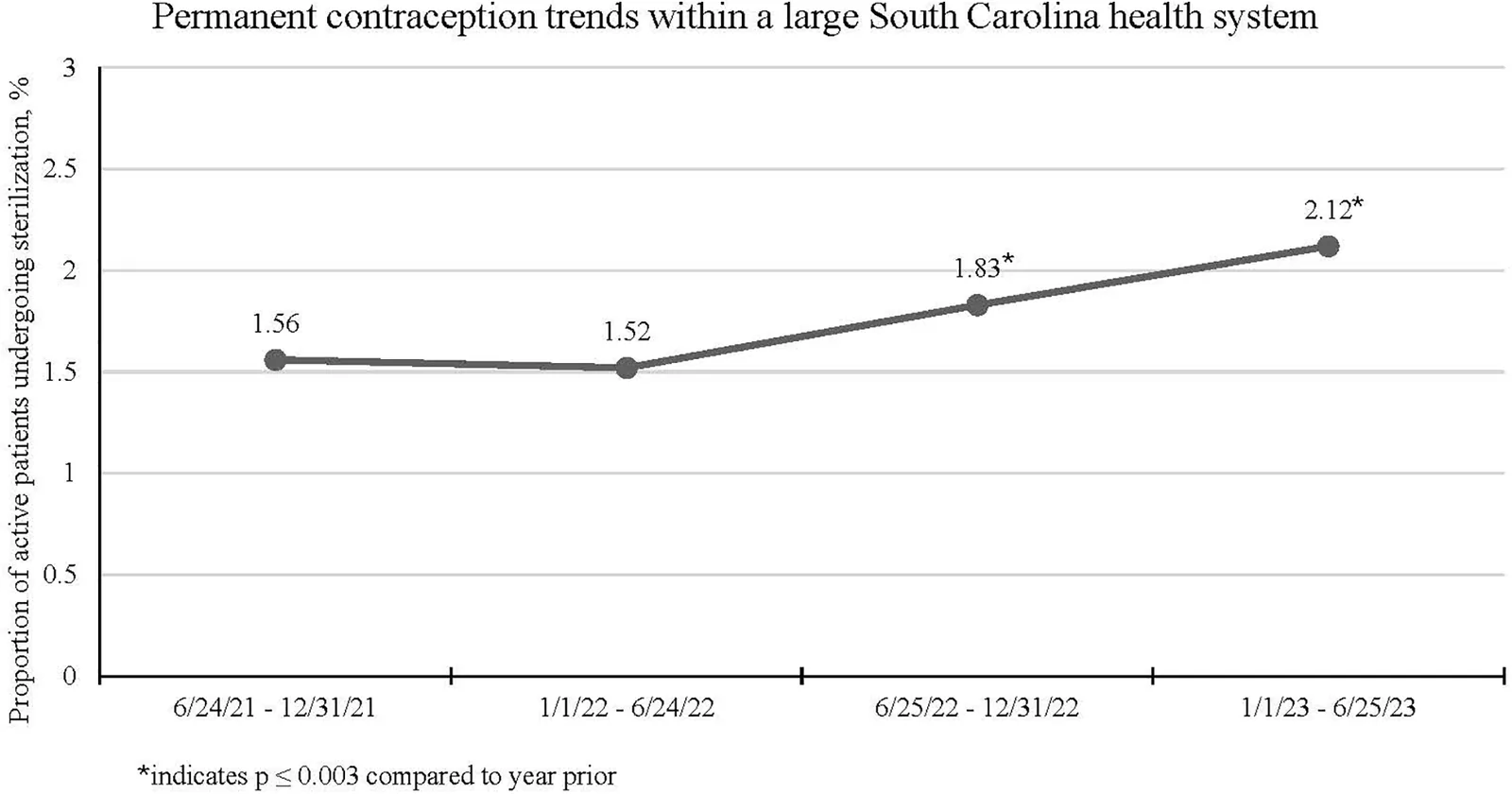
Proportion of active reproductive-age patients undergoing a sterilization procedure at a multi-hospital health system in South Carolina after the *Dobbs* decision.

Across the study period, age, race, ethnicity, marital status, healthcare payor, parity, and gravidity differed significantly between patients who did and did not undergo sterilization procedures (*p* < 0.0001). Patients undergoing sterilization procedures were younger [32.6 [5.5] vs. 34.4 [8.2]] and had a higher percentage of patients that were Hispanic/Latinx (11.8% vs. 5.9%), single (42.6% vs. 40.7%), and insured by a government healthcare payor (55.2% vs. 23.5%). A higher percentage of patients who did not undergo sterilization were Black (27.2% vs. 24.3%) or White (62.4% vs. 60.1%), married (52.6% vs. 49.8%), and had a commercial healthcare payor (71.0% vs. 43.4%) or were self-pay or uninsured (5.2% vs. 0.2%). Patients undergoing sterilization also had a higher parity (2.9 vs. 1.4) and gravidity (3.6 vs. 1.9) (Table 4).

**Table 4:** Demographic characteristics of reproductive-age patients who did and did not undergo a sterilization procedure at a multi-hospital health system in South Carolina (June 2021-June 2023).

Characteristic	All	Did not undergo sterilization procedure	Underwent sterilization procedure	*P*- value
	141,705	98.0 (138,799)	2.0 (2,906)	
Age, years, mean (SD)	34.4 (8.2)	34.4 (8.2)	32.6 (5.5)	<0.0001
Race/Ethnicity				<0.0001
Black	27.1 (37,738)	27.2 (37,039)	24.3 (699)	
Hispanic	6.0 (8,352)	5.9 (8,011)	11.8 (341)	
White	62.4 (86,712)	62.4 (84,979)	60.1 (1,733)	
Other	4.5 (6,249)	4.5 (6,140)	3.8 (109)	
Ethnicity, Hispanic	7.3 (10,194)	7.2 (9,772)	14.6 (422)	<0.0001
Marital Status				<0.0001
Single	40.7 (55,985)	40.7 (54,768)	42.6 (1,217)	
Married	52.5 (72,210)	52.6 (70,786)	49.8 (1,424)	
Life Partner	0.7 (987)	0.7 (943)	1.5 (44)	
Divorced	5.7 (7,797)	5.7 (7,637)	5.6 (160)	
Other	0.4 (511)	0.4 (498)	0.5 (13)	
Healthcare Payor				<0.0001
Commercial	70.4 (99,567)	71.0 (98,306)	43.4 (1,261)	
Government	24.1 (34,115)	23.5 (32,511)	55.2 (1,604)	
Self-Pay/Uninsured	5.1 (7,163)	5.2 (7,127)	0.2 (5)	
Other	0.4 (530)	0.4 (525)	1.2 (36)	
Parity, mean (SD)	1.4 (1.3)	1.4 (1.3)	2.9 (1.4)	<0.0001
Gravidity, mean (SD)	2.0 (1.8)	1.9 (1.8)	3.6 (1.9)	<0.0001

## 4 Discussion

### 4.1 Principal findings

This representative sample of patients in South Carolina demonstrated an increase in the proportion of sterilization procedures following the *Dobbs* decision on June 24, 2022.

### 4.2 Results

The increase in permanent contraception procedures corresponded to approximately 240 additional tubal sterilization procedures in the second half of 2022. This represents a larger increase in procedural volume than has been reported previously in other multicenter studies (4). While some states experienced a return to baseline utilization following an initial ‘post-*Dobbs’* spike (1), South Carolina continued to rise over the study period. This trend is consistent with national medical record data, which has demonstrated an increase in observed permanent contraception procedure rates nationwide amongst adults aged 18 to 30 immediately following *Dobbs* (5).

### 4.3 Clinical implications

These findings suggest an association between policies restricting access to abortion services and increased utilization of permanent contraception. Future research evaluating patient experiences would provide additional insight into individual factors influencing decisions regarding permanent contraception.

### 4.4 Research implications

Although beyond the scope of this study, further analyses could evaluate trends in sterilization utilization over time while adjusting for demographic characteristics to assess procedural volume on a monthly or yearly basis. Furthermore, there is opportunity to continue monitoring changes in sterilization utilization within the context of shifting political climates. Since the completion of this project, Title X funding for sterilization procedures has been terminated in South Carolina. The loss of funding will likely have more far-reaching consequences, including potential financial impact on patients requesting the procedure and healthcare systems in their decision regarding whether to continue with the procedure without funding.

### 4.5 Strengths and limitations

The strengths of this study include the large population size and the inclusion of patients from a substantial portion of South Carolina residents. As the state's largest nonprofit healthcare organization serving over1.4 million patients, this is a cross section of almost 25% of the South Carolina population (12).

Limitations include the challenges inherent to conducting retrospective analyses using large electronic health record datasets. Patients who underwent permanent sterilization before inclusion in the study could not be excluded. The study population also does not include patients receiving care from other healthcare institutions and patients who may be seeking care and are eligible but are unable to receive it. Some descriptive data was limited to reported categories within the electronic health record. For example, “single” would include those who are unmarried but partnered or cohabitating. Additionally, vasectomy rates were not evaluated, as other studies have done (10). Importantly, this study only evaluated outcomes through one year following *Dobbs*, and longer-term trends beyond this time remain unknown.

## 5 Conclusions

The proportion of patients undergoing tubal permanent contraception in South Carolina increased immediately following the *Dobbs* decision and continued to rise over the subsequent year.

## Data Availability

The raw data supporting the conclusions of this article will be made available by the authors, without undue reservation.

## Appendix A

**Table:** CPT and ICD-10 codes used to identify sterilization procedures among reproductive-age patients at a multi-hospital health system in South Carolina (June 2021-June 2023).

**CPT code**	**Procedure description**
58600	Ligation or transection of fallopian tube(s), abdominal or vaginal approach, unilateral or bilateral
58605	Ligation or transection of fallopian tube(s), abdominal or vaginal approach, postpartum, unilateral or bilateral, during the same hospitalization (separate procedure)
58611	Ligation or transection of fallopian tube(s) when done at the time of cesarean delivery or intra-abdominal surgery (not a separate procedure)
58670	Laparoscopy, surgical; with fulguration of oviducts (with or without transection)
58671	Laparoscopy, surgical; with occlusion of oviducts by device (e.g., band, clip, Falope ring)
58565	Hysteroscopy, surgical; with bilateral fallopian tube cannulation to induce occlusion by placement of permanent implants
58661	Laparoscopy, surgical; with removal of adnexal structures (partial or total oophorectomy and/or salpingectomy)
58700	Salpingectomy, complete or partial, unilateral or bilateral (separate procedure)

**ICD code**	**Diagnosis description**
Z30.2	Encounter for sterilization
Z30.09	Encounter for other general counseling and advice on contraception
Z30.8	Encounter for other contraceptive management
